# Understanding the unmet support needs of young and young adult carers and their families

**DOI:** 10.1371/journal.pone.0310766

**Published:** 2024-09-26

**Authors:** Nicola Brimblecombe, Madeleine Stevens, Sara Gowen, Robin Skyer, Jo Moriarty

**Affiliations:** 1 Care Policy and Evaluation Centre, London School of Economics and Political Science, London, United Kingdom; 2 Sheffield Young Carers, Sheffield, United Kingdom; 3 Sociology, Social Policy and Criminology, University of Southampton, Southampton, United Kingdom; 4 NIHR Health & Social Care Workforce Research Unit, King’s College London, London, United Kingdom; Faculty of Health Sciences - Universidade da Beira Interior, PORTUGAL

## Abstract

Support for children and young people who provide unpaid care is important to help prevent negative impacts of caregiving on their education, employment, mental health, and social relationships. We aimed to address an evidence gap about what services and support are needed from young carers’ perspectives. We carried out focus groups or in-depth interviews with 133 carers aged 9–25 in England. Expressed unmet need for services and support could be grouped in three categories: support that would reduce or remove young people’s need to provide care, help improve the lives of the people they care for, mitigate against impacts of providing care on their mental health, wellbeing, education, social participation and leisure activities, and, whilst they are still providing care, assist them in their caring role. Action is needed to address these currently unmet needs and implement young carers’ and their families’ rights to support.

## Introduction

Providing care can negatively impact on children and young people’s education, employment, mental health, and social participation [1–4] with associated costs to individuals, government, and the health service [1]. Support for young carers and the people they care for is therefore crucial and England, where our study was conducted, has brought in substantial new rights to support for young and young adult carers since 2014, albeit with limited and variable implementation of some of those rights [5]. England is now seen as relatively advanced in terms of young carer rights [6]. New rights include to a young carer needs assessment which should take into account and seek to address whether the young carer is providing ‘excessive’ or ‘inappropriate’ care; impacts of caring on the young person’s wellbeing, health, education or employment; and whether any of the young carer’s needs for support could be prevented by providing services to the person cared for [7, 8]. However, very little research has been carried out in this English rights context on how those rights best translate into how best to support young carers, address their unmet needs, and prevent and/or mitigate negative impacts, especially from the young carers’ own perspectives. Previous evidence suggests wide-ranging support gaps [9, 10]; however this research took place in a different young carer support and rights context to our study, either because of the period when the research took place or the country context. In light of the evidence gap, the policy and practice context, and the impacts on young and young adult carers of providing care, we conducted a study to investigate what support young and young adult carers found helpful or needed. The study overall aimed to investigate the perspectives of young and young adult carers on the helpfulness of support received and what prevented support from being helpful; and what support was needed or wanted that they did not already receive (unmet need). These two different aims corresponded to different segments of the data collection (focus groups and interviews with young carers) and were analysed separately. The results of the first analysis, published separately, gave a comprehensive picture of how best to support young carers by improving existing services [11]. This current paper addresses the question of unmet need, and the analysis was conducted based on a different type of data emerging from the same focus groups and interviews, as will be explained in the methods.

The conceptual framework for this paper drew on two frameworks that consider the different possible goals of carer support practice and policy: (i) Purcal et al (2012) and (ii) Twigg and Atkin (1994). (See also Table 1) Purcal et al’s framework applies specifically to young carer service support and postulates three main goals of such support: prevention, mitigation, and assistance [12]. ‘Prevention’ in Purcal et al.’s framework refers to services that aim to avoid the entrenchment of a young person’s caring role by adequate support to the care recipient *before* the child takes on the caring role or to achieve a reducing or removing care by the child if that is already taking place. In Twigg and Atkin’s [13] framework on whether and how the statutory care system engages with carers, prevention aligns most closely with the ‘superseded carer’ model which aims to replace unpaid care with other forms of support for the care recipient. This can be to ‘*ease the lot*’ of the carer, including by helping them to stop providing care, and/or to increase independence for the disabled person [13]. ‘Mitigation’ in Purcal’s framework refers to support that reduces negative impacts of caring upon the young carer. In Twigg’s categorisation, this ‘mitigation’ model corresponds to ‘carer as co-client’ with needs of their own to be supported. The third main possible goal is ‘assistance’. Similar to Twigg’s ‘carer as resource’ model, this approach sees unpaid care as an available and free source of care and seeks to sustain that care. Interventions will therefore aim at ensuring that carers are supported and sustained in their unpaid caring role through, for example, information and peer support.

**Table 1:** Guiding conceptual frameworks.

COMPONENTS	
Purcal et al (2021)	Twigg and Atkin (1994)
**‘Prevention’**: services that aim to avoid the entrenchment of a young person’s caring role by adequate support to the care recipient before the child takes on the caring role or to achieve reducing or removing care by the child if that is already taking place.	The **‘superseded carer’**: practice approach that aims to replace unpaid care with other forms of support for the care recipient.
**‘Mitigation’**: support to reduce negative impacts of caring upon the young carer	**‘Carer as co-client’**: practice approach that recognises carers have needs of their own to be supported.
**‘Assistance’**: support that helps with young carers’ caring role	**‘Carer as resource’**: practice approach that sees unpaid care as an available and free source of care and seeks to sustain that care.

Our research question was: ‘What do young and young adult carers aged 9–25 and the people they care for perceive to be their unmet needs for support?;?’ In line with standard definitions, we define carers as someone who looks after, or gives help or support to a family member, partner or friend who needs help because of their long-term physical or mental ill health, substance misuse, disability, and/or problems related to old age [14]. As is the case in this paper, young carers are usually defined as aged under 16 and young adult carers as aged 16–25. For shorthand, we mainly refer to both as ‘young carers’ throughout.

## Methods

Recruitment took place in four localities in England which differed by regional and geographical type and socio-demographic characteristics. Participants were invited to take part in focus groups with an option to take part in an interview instead. The inclusion criteria were being aged between 9 and 25 and providing unpaid care. Participants were recruited through young carer organisations, schools and colleges. We worked with partner organisations in each locality to facilitate recruitment. These organisations had extensive experience and expertise of engaging with young carers and care recipients historically less likely to access services in their areas, enabling us to reach a diverse range of participants. We held 19 focus groups in total, one online and the remainder in person, and eight online or phone interviews. The resultant sample was 133 participants with a range of caring and life circumstances and socio-demographic characteristics. Forty were aged 9–11, 57 aged 12–15 and 36 aged 16–25; most of the latter were at the lower end of this range. Where we have information, 25 cared for a sibling, 46 for a parent and two for another relative. Some cared for more than one person. The people cared for had a range of physical and/or mental care and support needs.

Focus groups and interviews took place between 1/6/2021 and 31/5/2022. Focus groups were co-facilitated by the research team and local project workers. Informed consent or assent was sought from all participants and parental consent for children 15 or younger. Consent and assent were given in writing, except for online or phone interviews where verbal consent was given which was audio-recorded and noted on a paper copy of the consent form. Focus groups and interviews were guided by a topic guide, audio-recorded with participants’ permission, and fieldnotes taken. Focus group recordings were transcribed where possible; in many cases multiple voices and discussions whilst the activity was going on made this impossible. In-person focus groups were held in venues familiar to participants such as a young carer or local community centre Ethical approval was granted by LSE Ethics on 21 May 2021 (Ref. 1247).

We used a range of methods in the focus groups, co-developed with practitioners and young carers and informed by the available literature [15]. This facilitated an informal atmosphere and enabled a range of ways (verbal, visual, written, group, individual) for young carers to express unmet need, gaps in support, and preferred options for support. The results reported in this paper are part of a larger study which looked at what was helpful about existing support what could be improved [11], using a different segment of and methods within the focus group or interview to this current paper. This current paper reports on an exercise where participants—either individually or with the research team—were guided to annotate a simple representation of a young carer and the person they care for with their views of the support they or someone else in their situation would receive in an ‘ideal world’, and which currently unmet needs it would help meet. Specifically, they were asked to consider what sort of things would help support young people who look after someone at home. We explained that this could include both help for the young carer and for the person they care for. Researchers discussed the activity with the young people whilst they were doing it, asked prompts, and took notes. In some cases, participants expressed a need for a service or support they knew existed because of information provided by the school or young carers organisations, for example, or discussion with other young carers including in earlier parts of the focus group. In other cases, participants described the support that would meet their needs regardless of knowing whether it existed, or they would describe the need it would meet.

To enable an informal atmosphere and safe space, we took advice from the study’s young carer advisors and from practitioners with expertise working with young carers. Support was provided by experienced young carer support workers. At the beginning of each group, ground rules for participants, support staff and researchers, such as keeping confidentiality within the group, listening and being respectful, were jointly discussed and agreed. In the focus group, we began with support worker-led ‘icebreakers’ and ended with a ‘cool down’ activity, both unrelated to the study topics. We then reminded participants of the information sheet with helplines as well as the availability of the research team and the attending support workers after the group. Focus groups lasted between 2 and 2.5 hours including time for food and breaks, icebreaker and cool down. Of the remaining time, activities overlapped but the exercise reported in this paper was one of three main activities/topics and made up approximately a quarter of the data collection time in the focus groups.

The overall approach for the analysis was reflexive thematic analysis [16], incorporating both deductive and inductive elements. This began with familiarisation with the data, including reading and reviewing annotated pictures, transcripts, and notes. An initial coding framework was then developed, structured around the conceptual frameworks [12, 13] and the research question: ‘‘What do young and young adult carers aged 9–25 and the people they care for perceive to be their unmet needs for support?. The initial coding frame thus had the following overarching themes: reduction or removal of caring (prevention or superseded carer); mitigation of negative impacts (mitigate or carer as co-client); assist in caring role (assistance or carer as resource).

We then gathered all data relevant to each theme. Themes were adapted in response to the data. For example, in Purcal *et al*, ‘mitigation’ services include respite or a break from caring. In our study, it became apparent during analysis that a break from caring provided more of a ‘prevention’ function by reducing the care provided. As a second example, mitigation in the original framework includes reducing negative impacts of caring; during analysis, ‘improving outcomes’ was identified as an addition to the ‘mitigation’ category. We also identified sub-themes within each initial themes. For example, the initial coding frame included ‘remove or reduce caring responsibilities’ as a theme; sub-themes included ‘to enable time and energy to pursue personal goals and interests’ and ‘mode of addressing need’. A further example was the sub-theme of awareness, recognition and understanding of young caring and of disability and mental ill health as an assistance to providing care; this aspect was not in existing frameworks. Themes and sub-themes were iteratively reviewed, informed by discussions on initial themes and sub-themes with young carer and practitioner advisors. NVivo12 software was used to organise data.

## Results

Drawing on both Purcal’s and Twigg’s frameworks, the findings about what support was perceived as needed by young and young adult carers and what needs such support would meet are structured around the three main themes that conceptualise types of currently unmet support needs: (i) prevent, reduce or remove caring responsibilities (the superseded carer); (ii) mitigate the effects of providing care and, going beyond that, improve the affected aspects of young carers lives (carer as co-client); and (iii) assist the carer in their caring role (carer as resource). (See Table 2.) Unless stated otherwise, quotes included in the findings represent annotations from young carers on the simple representation of a young carer and the person they care for. All quotes are anonymised.

**Table 2:** Unmet support needs: Themes and sub-themes.

THEMES	SUB-THEMES
Support that reduces young carers’ caring responsibilities (prevention)	To enable young carers to pursue their own personal goals and interests
	To have less or shared responsibility
	To improve the life of the care recipient
	Mode of addressing ‘reduce or remove’ needs
Support that helps with some of the impacts of caring and/or other life issues and/or helps improve outcomes in those domains (mitigation)	Mitigating mental health and wellbeing impacts and improving mental health
	Mitigating educational impacts and improving educational outcomes
	Mitigating social connection and relationships and facilitating ability to make connections and improve relationships
Support that helps with young carers’ caring role (assistance)	Peer support
	Information and advice
	Recognition, understanding, and awareness

### Prevention: Young carers and their families say they need support that reduces young people’s caring responsibilities

Participants identified three main needs that required the reduction or removal of caring responsibilities. These needs, or sub-themes, were: (i) to enable them to pursue their own personal goals and interests; (ii) to have less or shared responsibility; (iii) to improve the life of the care recipient; and (iv) mode of addressing ‘reduce or remove’ needs.

#### Pursuing personal goals and interests

Reductions in young carers’ care provision would mean more time, but also more physical and emotional energy, for the young person to pursue personal goals and interests. This included education. Having someone to check on the cared for person while the young person was at school, for example, meant they could attend school and concentrate whilst there and/or could concentrate on school or college homework. Having support for the person at home would mean the carer could go to after school clubs, and many expressed an interest in pursuing activities and hobbies. Less caring would also enable greater social participation and connection. This could be with non-cared for family members but was mainly with friends. For example:

‘*Someone in the house (I can go out to the park)*’

‘*Someone to sit with my mum while I go out with my friends*’

A further goal identified by participants that a reduction in care could address was to have some time to and for themselves. This was described by one young carer as ‘*to be able to do what I like when I want without worrying about my dad*’. This could be on a regular basis or for specific activities, for example, to have some ‘*space away*’, *or a* ‘*get-away/residential so that I can relax & know they’re safe but also relax myself*’.

#### Shared or less responsibility

A reduction or removal of care would reduce the responsibility felt by the young carers in our study, something that caused a great deal of anxiety and stress. One young carer described the role this reduction would meet as follows: ‘*outside help [would] ease my load of work and mental health*’. Care in an emergency–’*a person to call when parent is sick*’ ‘*support if needed 24/7*’—was an aspect of this.

#### Improving the life of the care recipient

Mental or physical health support for the person they cared for to prevent that person being physically and/or mentally unwell, or improve their health, was a key unmet need identified by the young carer. This would help by reducing their caring role and the stresses and strains of living with someone with, for example, mental ill health. It was also strongly related to a desire for the person they cared for to feel happier, or in less physical pain:

‘*Seeing my mum happy and energetic all the time would give me a lot of motivation to be happier*’.

‘*Would like my sister to walk and talk > be good if she has people to help her do this*’.

#### Mode of addressing ‘reduce or remove’ needs

In describing the type of services and support needed to bring about a reduction of their physical and/or emotional caring, the young people identified several sources of alternative support for the care recipient, although in a number of cases young people did not know where the support would come from, only that they needed more help and to provide less care. For example, ‘*people coming to help*’, ‘*extra support for her needs*’. Where identified, mode of reducing care by young carers included social care services such as paid careworkers, respite care, aids and adaptations, mental health support, activities outside the home, or help from family.

As the following annotations by young carers show, reducing care by young carers could come via social care services to help with personal care (‘*carers—wash hair*’); supervision (‘*having contact every day to make sure she doesn’t black out and fall*’); or support to enable the care recipient to leave the house (‘*a care worker to take her out and understand her more*’).

Increased accessibility within the house and in the external environment for the person with care needs through, for example, aids and adaptations, were also key to reducing young caring, as well as increasing independence for the care recipient. This could be (more affordable) specialist equipment such as wheelchairs, alarms, or it could be street or home adaptations. Some examples are to ‘*fix the high kerbs*’ or have ‘*house adaptations for physical/ mobility needs*’.

Mental health support was an important part of what was needed to reduce or replace often intensive emotional care by the young carer:

‘*More help for my mum so I don’t have to take all the emotional baggage*’

Help to deal with addiction and substance misuse was also mentioned by young carers:

‘*Regular group + people to talk to help her stop drinking*’

‘*Help support with drug/alcohol abuse*’.

Young carers also expressed a need for practical support to reduce their domestic responsibilities and time spent on housework, shopping, and care of younger siblings. This included:

‘*Outside help for cleaning/chores to ease my parents*’ *pains and illnesses*’

‘*Somebody to look after my sister while I care for my mum*.’

Replacing or superseding the young carer, giving them a break, and improving the life of the care recipient could also come about, in part at least, by improving the social connectedness of the care recipient and their ability to participate in activities outside of the house. In the views of young carers, this included ‘*trips for the cared for person*’ and ‘*support to go outside*’.

Work was another component of participation in activities outside of the house as expressed by young carers—’*help her to get her job*’.

Support did not necessarily need to come solely through statutory services, or even through statutory services at all. Alongside the need for social and community connection and emotional support for the care recipient, many young people expressed a need for more help from other family members:

‘*More family members to help*’

‘*Dad should help*’

‘*If my brother helped out a bit more*’ (Young carer interview)

### Mitigation: Young carers say they need support that helps with some of the impacts of caring and/or their other life issues

‘Mitigation’ involves services and support for young carers that aim to address, and in our framework to also improve, some of the areas of young carers’ lives that caring has negatively impacted. Sub-themes identified by young carers were: support for their mental health and wellbeing, education, and social connections and relationships. For some participants, these appeared to be areas where unpaid caring had had negative effects, education being perhaps the clearest example. In others, there was a more complex mix of care provision exacerbating issues that existed for the young person outside of their caring role, for example their mental health. This highlights the importance of recognising the context in which young caring takes place for individuals and supporting them accordingly.

#### Mitigating mental health and wellbeing impacts

Support for their mental health, wellbeing, and stress levels was an aspect of their lives that many young carers mentioned in their annotations, as the following examples show:

‘*Stress release like to ring someone and after school*’.

A ‘*mental health service that is provided to young carers to help them express home struggles and worries*’.

Mental health support for young people could come from National Health Service (NHS) or voluntary or community sector mental health services. It could also come from school counselling services and/or from young carer organisations. A frequently mentioned gap in support was someone available to talk to, as the following quotes show:

‘*Have someone to talk to about my situation*’.

‘*Someone who understands properly what happens at home*.’.

Not all young people wanted someone to talk to:

‘*It could be good for other people*, *but I don*’*t really like to express what*’*s happening*. *I just like to keep it to myself*’.

Schools, as well as mental health services and young carer organisations, were often mentioned as a place where they would like someone to talk to:

‘*It would help if I could speak to someone at school about what*’*s happening at home*’.

#### Mitigating educational impacts

Another domain where ‘mitigate and improve’ support was needed was for young carers’ education. This could be help with their schoolwork and homework, especially where parents could not help or the young person had no time or space to do it at home. Or it could be more flexibility over homework as the following two young carer annotations show: ‘*longer homework dates if teacher noticed something isn*’*t right*.’; ‘*less homework for young carers or a better understanding of home life*’. However, young carers were aware that there was a difficult balance for schools between mitigating the effects on young carers’ stress and mental health and on their educational attainment.

#### Mitigating social connection and relationships

Social connection and ability to participate socially was often disrupted in young carers’ lives in our study and many expressed a need for help with making friends and for helping friends be more understanding of their situation. One way to facilitate development of friendships is through removing or reducing their caring role, as described above. It could also be part of mitigatory support. Young carers talked about the need to ‘*meet new friends*’ and ‘*have friends that understand*’. Family relationships can also be disrupted by caring in the family and young carers expressed the need for better relationships with family as well. For example, ‘*free family therapy to work out rules*, *because the parental relationship often flips*’.

### Assistance: Young carers say they need support that assists with their caring role

‘Carer as resource’ support models and policies aim to assist the carer in their caring role, including ensuring that it is sustainable. For young carers in our study, sub-themes related to unmet need for assistance included peer support; information; and awareness, recognition and understanding.

#### Peer support

This was a key unmet need sub-theme for young carers in our study. Peer support may have a particular salience for young carers because of the importance of peer support to children and young people in general [e.g. 17] and because their non-carer friends sometimes found it difficult to understand about their caring or the disability or illness of the person they cared for. Also, because of fear of disclosure of private and potentially sensitive family or personal information to their peers who did not have similar experiences. ‘*Meeting others like myself*’, ‘*friends who are carers*’ and ‘*people who are in a similar situation who can understand*’ would mean they didn’t have to explain themselves and potentially be judged, a main perceived benefit of peer support. Participants explained how peer support could be within a voluntary sector young carer group or within school, either as a regular in- or after-school group or simply to ‘*know other young carers at school*’.

#### Information and advice

Information and advice was another aspect of support that young carers felt might assist them in their caring role. This included information about the care recipient’s care needs, how needs might change, and how best to support them, as the following examples show:

‘*Info on situations/conditions*’.

‘*Lessons to help you with the person you care for*!’.

As the young carers describe below, being kept informed about the care recipient’s treatment would also help the young person in their caring role:

‘*Being able to know my mum is safe when she is in hospital*’.

‘*For the doctor or someone to talk to me about my mum*’*s health*.’.

A further gap was information about support for young carers.

#### Recognition, understanding, and awareness

Recognition, understanding and awareness of young caring, as well as inclusivity within services, were additional unmet needs. Young people felt that these qualities could assist them in their caring role. This meant their views being believed and respected:

‘*Being able to tell someone that you think someone needs to be looked after*’.

‘*Other people shouldn*’*t tell me what to do–we are the experts who know the people we care for*’.

Young carers also felt that greater recognition, awareness and understanding of young carers, including how caring affected young people, was needed in schools and other services. This included:

‘*School know I*’*m a young carer and share it with the right people*’.

It also meant the school understanding what young caring involves and what support young carers might need, as the following example illustrates:

‘*Schools being informed about carers & how to help them*’.

Moreover, it was felt that schools had a role to play in increasing awareness and understanding among their students:

‘*I used to have some friends a couple of months ago where I used to say to them*, *I care for my brother*, *but they don*’*t really know full on what I do for my caring role and they used to pick on me…*.*I kind of wish they take it further in school like they talk about young carers in school*’(Young carer quote from discussion).

Wider public awareness and understanding about young carers and about people with health conditions and disabilities was another unmet need identified by young carers and care recipients in our study. Reducing this could help in young people’s caring role. The young carers explained this as follows:

‘*More people understanding that people are young carers*’.

‘*People to understand and appreciate people that have disabilities*’.

### Prevent, mitigate and assist? Multiple roles

Support or services for young carers may need to have multiple functions, supporting both the young carer and care recipient, reducing, mitigating, and/or assisting the caring role. The support that several young carers described as needed could be described as being multi-function and ‘whole family’: providing help for the person they care for (and so reducing their caring role and improving the life of the care recipient) and help for themselves. The following quotes are some examples:

‘*This place can help you and the person you care for!*’.

‘*Help us as well as helping him*’.

‘*I don*’*t think it should be just me who gets to speak to somebody*. *I feel like maybe my mum could be able to speak to someone too*’ (young carer quote from discussion).

## Discussion

Our study, with a wide range of young carers across England, adds to theory and evidence in four main ways. First, our findings add to the previously sparse research about what support is perceived as needed by young carers. Specifically, we gathered perspectives on needed support in a relatively advanced support and rights context (England, [6]); meaning we were able to ascertain what were gaps in implementation and what were gaps in policy. This distinction is key to improving young carer support. Second, we investigated the range of support that might be needed. Previous research has tended to focus on specific types of support need; however young carers and the people they support may have multiple needs and areas of their lives that are affected by providing care [1–3, 18]. Our study facilitated young carers to consider a wide range of possible carer-related support that might contribute to making their lives easier and as a result showed the multiplicity of support that is needed.

Third, we build on existing theory. As described in the paper, we made several adaptations to the Purcal framework in response to the views of young carers on what support they need and what roles it would fulfil. These include categorising breaks from caring as ‘prevention’ rather than assistance and the addition to the ‘assistance’ element of the typology of better awareness, recognition and understanding of young caring and of disability and mental ill health. Additionally, research to date on unmet need has generally only considered the perspective of people with care needs [19, 20]. Our study, showing that unmet need for care and support for the person with care needs can also be an unmet need for others in that relationship further makes the case for a conceptualisation of and research on unmet need to take a carer-inclusive, i.e. dyadic, approach in order to get a full picture of unmet need, how to address it, and the enablers and constrainers of addressing it [21]. For an even fuller picture, two other elements are important. One is the practitioners responsible for providing and delivering support who can help address unmet need but also may be impacted by it [22] and have their own constraints and enablers–a triadic approach may therefore be more useful. The other is the context in which caring and unmet need takes place, which includes the policy, funding, and societal context. The resultant ‘contextual triadic approach’ can be used to understand and address unmet need for support and, more widely, to other care need and caring situations.

In the past decade, England has seen advances in policy and practice towards young carers and we might have expected to see this reflected in our findings. However, we found that many of the same gaps in support remain. One example is our finding of the expressed need, currently unmet, for more mental, emotional, physical and practical support for the care recipient. More support for the care recipient or less caring, was seen in previous research in time or country contexts where there are, or were, fewer rights to support for young carers [10, 23]. Since that research there has been legislation in England that explicitly includes provision for services for the care recipient to support the young carer. Our findings, showing this unmet need persists, point to failures of implementation. Because some young carers in our study did receive services for the person they cared for [11], implementation appears to occur but be uneven.

In terms of mitigation of negative effects of caring and improvement of outcomes, young carers in our study expressed the need, currently unmet, for support with their mental health, emotional wellbeing, and education. This is consistent with previous research [10, 24]. We also found that young people wanted support that facilitated greater social connection for themselves; better relationships with family; and ability to take part in hobbies and activities. In our study, support to help them in their caring role–assistance–included support from peers with similar experiences and information and advice about the care recipient’s mental or physical health condition as some previous research has also shown [23, 25]. Again, that this support is still lacking despite policy and practice developments over the last two decades in England shows the entrenched and complex nature of unmet need for support among young carers. A further form of assistance identified in our study, which could be considered an addition to the Purcal *et al*. (2012) framework, was greater multi-agency awareness, recognition and understanding of young carers and disabled people and people with long-term mental or physical ill health.

Services and support for carers may have multiple functions, and unmet needs to be addressed can be multi-faceted. For those that are already providing care, such as the young people in our study, a reduction or removal of their caring responsibility may be needed to enable them to pursue personal goals and interests and prevent future negative impacts in conjunction with mitigatory actions to redress the impacts already incurred. Similarly, mitigation can take place alongside ‘assistive’ actions to help support the carer in their caring role. However, the assist function of services, or ‘carer as resource’, implicitly involves sustaining the caring role rather than preventing or removing it [13]. For many service providers, individuals and families, supporting the young carer in their caring role, addressing the very immediate needs of children and young people who have entered a caring role, and mitigating negative consequences may be a pragmatic response to the consequences of the care recipient’s needs not being met in other ways. Purcal *et al*. also make the point that whilst the most desirable service goal is prevention of young caring through adequate formal support for people with a chronic illness or disability, ‘*mitigation and assistance-type services…can and do provide important relief and in some cases may be all that is required to prevent negative outcomes for the young carer*’ (Purcal et al., 2012, p. 10).

### Strengths and limitations

A strength of our study is that we talked in depth to over 130 young and young adult carers of different ages, gender, ethnicity, socio-economic status, geographic location and home and caring circumstances whose voices and opinions are not often heard. We took various steps, with advice from our advisory groups, to ensure they felt comfortable speaking, were included, and their voices heard and understood. This enabled us to gain a rich and deep understanding of what support is needed and valued from a diversity of perspectives. However, a potential limitation is that some voices are still missing. For example, some particularly marginalised group such Roma and Traveller, asylum seeker or migrant young carers. Future research, which would ideally be peer-led, could investigate the extent to which there are additional unmet support needs for these young carers. Another possible limitation is that we mainly recruited through young carers organisations. However, this was balanced by recruitment through schools and extensive outreach and engagement by the collaborating organisations prior to our project starting. Focus groups and interviews can be subject to social desirability and/or researcher bias. We took a number of measures to mitigate this, including individualised and anonymous ways of contributing and reiterating that there are no wrong answers; creating a safe and informal sharing space; and being reflexive in our practice both in the focus groups and in the analysis.

### Policy implications

Our findings of the extensive unmet needs of young carers and the person they care for, and the range of support needed, has fundamental implications for policy and society about how we view children and young people providing care as well as how we best support them. It has implications for overall funding and how and when budgets are allocated and spent. For example, preventative services may be in different budget domains, both locally and nationally, than mitigatory services. Preventative services will incur costs before caring has started or become entrenched; mitigation and assistance will incur those costs at a later stage. The broader context in which care is provided is also relevant. For example, young carer’s desire to pursue hobbies, activities and interests can be aided by reducing their care responsibilities (prevention), so they have more time, energy and focus and less anxiety to attend. However, young carers may also not be able to access support for financial reasons. Improving carer and disability welfare benefits as well as more accessible employment would help families with a young carer in them [26, 27]. Young carers and their families have significantly lower income than families with no young carer [28] and young carers are more likely to experience child poverty than their non-caring peers [29]. Lastly for policy and practice, our study suggests a wide range of ways in which support to address unmet needs could be provided. This included statutory care services for the care recipient, and also voluntary sector carer organisations, community and youth groups, schools, youth mental health services, family, friends, and peers.

## Conclusion

Young and young adult carers expressed unmet need for services and support that would reduce or remove their need to provide care, improve the lives of the people they care for, mitigate against some of the impacts of providing care on their mental health, wellbeing, education, social participation and leisure activities; and, whilst they are still providing care, assist them in their caring role. Taking into account context of providing care and unmet need is also important. Rights to reduction or removal of caring responsibilities, prevention of caring and of impacts, mitigation and assistance are all present in current legislation but failures of implementation are resulting in persistent and uneven unmet need for support.
